# *Lonicerae Japonicae Flos* as a Dietary Natural Product: Bioactive Constituents, Gut Health Modulation, and Therapeutic Potential in Ulcerative Colitis and Colorectal Cancer

**DOI:** 10.1002/fsn3.72366

**Published:** 2026-09-17

**Authors:** Xiaoyu Huang, Letian Zhang, Zenghui Shi, Ni Zhu, Yifei Liu, Yanhong Zhou

**Affiliations:** ^1^ School of Pharmacy Xianning Medical College, Hubei University of Science and Technology Xianning Hubei China; ^2^ School of Stomatology and Ophthalmology Xianning Medical College, Hubei University of Science and Technology Xianning Hubei China

**Keywords:** bioactive constituents, colorectal cancer, functional foods, gut health, *Lonicerae Japonicae Flos*, ulcerative colitis

## Abstract

*Lonicerae Japonicae Flos* (LJF), a traditional Chinese herb with applications in both medicine and nutrition, has emerged as a promising functional food ingredient owing to its diverse bioactive constituents and associated health‐promoting properties. This review systematically describes its botany, phytochemistry, and pharmacological activities, with particular emphasis on the roles of its principal constituents in ulcerative colitis and colorectal cancer and their implications for dietary interventions. LJF is abundant in phenolic acids and flavonoids, which exert pronounced anti‐inflammatory, antioxidant, and immunomodulatory effects through the modulation of key signaling cascades, including the NF‐κB and MAPK pathways, thereby attenuating oxidative stress, suppressing proinflammatory mediators, and preserving intestinal barrier integrity and gut microbiota homeostasis. Moreover, these constituents exhibit multiple effects against colorectal cancer, including the induction of apoptosis, inhibition of tumor cell proliferation, and suppression of angiogenesis and metastatic progression via the regulation of oncogenic signaling networks. Nevertheless, the broader application of LJF remains constrained by chemical complexity, insufficient mechanistic elucidation, and a paucity of robust clinical evidence. Future investigations integrating multiomics strategies, advanced food processing and delivery systems, and rigorously designed clinical studies are warranted to increase its translational potential in intestinal health management.

## Graphical abstract

Bioactive constituents, mechanisms of action, and potential applications of *Lonicerae Japonicae Flos* as a dietary natural product for promoting gut health and managing ulcerative colitis and colorectal cancer.

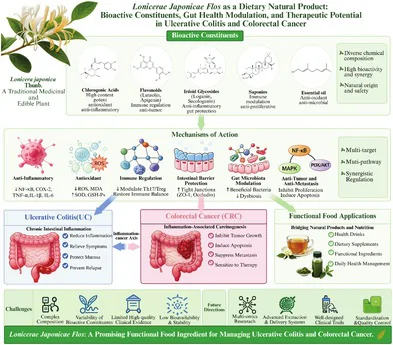

## 1 Introduction

Ulcerative colitis (UC) is a chronic, nonspecific inflammatory disorder of the colon characterized by continuous mucosal and submucosal inflammation that typically originates in the rectum and gradually extends proximally (Le Berre et al. 2023; Ordás et al. 2012). Its pathogenesis is multifactorial and involves complex interactions among genetic, immune, environmental, and infectious factors (de Dombal et al. 1969; Wangchuk et al. 2024; West 1971). Individuals with a family history of the disease, long‐term smoking history, or a preference for high‐fat diets are at a relatively high risk of developing UC. Nevertheless, the overall mortality rate of UC remains low. Over the past several decades, the incidence of colitis has shown a continuous upward trend, making UC a growing global health concern. Clinical studies have demonstrated that the incidence of colorectal cancer (CRC) in patients with UC is significantly greater than that in the general population. UC and CRC are linked through a continuous inflammation‐to‐carcinogenesis cascade, wherein CRC arising from inflammatory bowel disease is referred to as colitis‐associated colorectal cancer (CAC) and is one of the most severe complications of UC (Shah and Itzkowitz 2022). This pathogenic process is multifactorial and highly intricate, with prolonged chronic inflammatory stimuli, persistent oxidative stress, immune dysregulation, and disruption of the mucosal barrier collectively precipitating DNA damage and genetic mutations in epithelial cells. The sustained inflammatory milieu further predisposes the intestinal epithelium to dysplasia and malignant transformation (Jin et al. 2025). In recent years, the incidence and mortality of CRC have steadily increased, driven in part by population aging and lifestyle changes, including high‐fat diets, sedentary behavior, and rising obesity rates. CRC is characterized by considerable genetic heterogeneity, and its initiation and progression typically involve a multistep transformation from normal glandular epithelium to adenoma and ultimately to adenocarcinoma, accompanied by genetic mutations, epigenetic modifications, and immune‐inflammatory responses that include multiple molecular pathways.

Identifying safer and more effective therapeutic agents for the treatment of UC and CRC is highly important to society. Traditional Chinese medicine (TCM), as an integral component of Chinese medical practice, has notable advantages in healthcare owing to its diverse sources, controllable quality, and relatively low cost (Tang et al. 2008). TCM makes extensive use of plant, animal, and mineral resources, thereby providing a broad spectrum of therapeutic options. With advances in modern technology, TCM quality control has substantially improved, ensuring both the consistency and efficacy of medicinal materials. Moreover, the low cost of TCM not only increases its economic competitiveness but also facilitates the dissemination of TCM culture, thereby contributing to public health. Although conventional Western therapies for UC and CRC can alleviate symptoms or inhibit tumor growth, they are often limited by toxicity, adverse effects, drug resistance, and the high cost of biologic agents. In contrast, TCM has a long history in managing these diseases, with herbal medicines offering abundant resources and more cost‐effective alternatives to biologics and monoclonal antibody therapies. Commonly used herbal medicines for treating UC include *Lonicerae Japonicae Flos* (LJF), berberine, *Scutellaria baicalensis*, and *Curcuma longa* (Sun et al. 2023; Xu et al. 2024). In addition, classic herbal formulas such as Gegen Qinlian decoction and Banxia Xiexin decoction have been applied in the treatment of UC (Luo et al. 2024; Wang et al. 2023). In China, the use of TCM for treating UC dates back thousands of years and has shown distinct therapeutic advantages (Liu et al. 2022; Zhang et al. 2013). Numerous Chinese herbal medicines and their bioactive constituents have been demonstrated to have beneficial effects, including antioxidant, anti‐inflammatory, immunomodulatory, and antibacterial effects (Wang, Yin, et al. 2024; Yang, Luo, et al. 2021; Zhou et al. 2016). Furthermore, these herbal medicines can significantly alleviate UC‐induced colonic injury, reduce the disease activity index, improve mucosal immune function, and modulate the composition and activity of the gut microbiota (Lv et al. 2019; Ma et al. 2023; Wei et al. 2021; Zhang, Miao, Wang, et al. 2024). Accumulating evidence has also shown that several Chinese medicinal herbs, such as licorice, *Astragalus membranaceus*, *Atractylodes macrocephala*, ginseng, and *Codonopsis pilosula* , have shown potential to prevent and treat CRC (Li, Wen, et al. 2025; Lu et al. 2025). Herbal formulas including Sijunzi decoction, Huangqin decoction, and Shengjiang Xiexin decoction have likewise been used in the prevention and treatment of CRC (Liu, Zhang, et al. 2025; Shang et al. 2023). LJF, a well‐known traditional Chinese medicinal herb, was first recorded in Shennong Bencao Jing and is classified as a superior‐grade medicinal plant (Zheng et al. 2022). LJF is regarded as an herb of with both medicinal and nutritional value, functioning both as a conventional nutritional ingredient and as a bioactive agent with diverse pharmacological properties. The medicinal value of LJF is attributed primarily to its rich chemical composition. Modern scientific studies have demonstrated that LJF contains large amounts of organic acids, volatile oils, flavonoids, iridoids, triterpenes, and triterpenoid saponins, which confer a wide range of pharmacological activities, including anti‐inflammatory, antipyretic, antitumor, antibacterial, antiviral, antioxidant, hepatoprotective, lipid‐lowering, central nervous system–stimulating, and immunoregulatory effects (Zheng et al. 2022).

## 2 Materials and Methods

A comprehensive literature search was performed using several electronic databases, including PubMed, Web of Science, Elsevier, ScienceDirect, Scopus, and CNKI, to identify relevant studies published between 1969 and 2026. The search strategy included keywords such as LJF, LJF‐derived constituents, chlorogenic acid (CGA), luteolin (Lut), quercetin (Que), triterpenoid saponins, polysaccharides, ulcerative colitis, and colorectal cancer. We referenced the World Flora Online database (http://www.worldfloraonline.org) to confirm the scientific nomenclature of this plant. Bioactive components of LJF were systematically screened using the HERB platform, the Traditional Chinese Medicine Systems Pharmacology database (TCMSP), the Encyclopedia of Traditional Chinese Medicine (ETCM), and the Bioinformatics Analysis Tool for Molecular mechANism of Traditional Chinese Medicine (BATMAN‐TCM). Supplementary physicochemical and structural information was retrieved from the PubChem and Chemical Book databases. The chemical structures of the bioactive constituents were generated using ChemDraw, and the schematic diagrams were prepared with BioRender. The selection of studies was based on relevance, methodological rigor, and recency to ensure a comprehensive and accurate synthesis of the literature.

## 3 Clinical Manifestations and Therapeutic Approaches for UC

The distribution of UC incidence is bimodal, with a primary peak occurring between 15 and 30 years of age and a secondary, smaller peak occurring between 50 and 70 years of age (Ordás et al. 2012). As of 2023, epidemiological data suggest that UC affects an estimated 5 million individuals worldwide, with its incidence demonstrating a sustained upward trajectory globally (Le Berre et al. 2023). Clinically, UC is characterized mainly by diarrhea and abdominal pain, and in severe cases, hematochezia can occur. The major pathological features of UC include chronic inflammation of the intestinal mucosa, ulcer formation, epithelial damage, and abnormal mucosal repair (Kobayashi et al. 2020). The diagnosis of UC generally relies on colonoscopy, supplemented by various imaging modalities such as computed tomography (CT), magnetic resonance imaging (MRI), barium enema, and X‐ray examinations (Wangchuk et al. 2024). The treatment of UC depends on disease severity, with commonly used anti‐inflammatory agents including sulfasalazine and mesalazine (Nguyen et al. 2018). For patients with more severe disease, immunosuppressive agents such as corticosteroids, azathioprine, and cyclosporine may be administered. If the above treatments are ineffective, biologic therapies such as infliximab, a tumor necrosis factor (TNF) inhibitor, may be employed. In severe cases, colectomy may be needed. Researchers have clearly indicated that the etiology of UC is multifactorial and involves genetic predisposition, environmental factors, immune system dysregulation, and alterations in the gut microbiota (Shanahan 1993). According to contemporary TCM theory, the pathogenesis of UC and its progression to CAC is complex and characterized by the coexistence of deficiency and excess as well as the interplay among pathogenic and protective factors. This manifests as an underlying spleen deficiency accompanied by internal accumulation of damp–heat, often compounded by blood stasis obstructing the intestinal collaterals. TCM prevention and treatment strategies emphasize strengthening the spleen and replenishing qi to consolidate the body while clearing heat, detoxifying, resolving dampness, and removing stasis to address the manifestations, with flexible formulations tailored to syndrome patterns through the combination of heat‐clearing and detoxifying, qi‐tonifying and spleen‐strengthening, and, when necessary, blood‐activating and stasis‐resolving herbs. Recent research has indicated that such multicomponent prescriptions may modulate immune function, suppress intestinal inflammation, and restore gut homeostasis, thereby not only alleviating UC disease activity but also potentially reducing the risk of chronic inflammation progressing toward malignant transformation.

## 4 Clinical Manifestations and Therapeutic Approaches for CRC

CRC is characterized by malignant tumors that originate in the colon and rectum and is referred to as large bowel cancer. It represents a major global public health concern and is currently the third most diagnosed cancer and the second leading cause of cancer‐related death worldwide, with both incidence and mortality rates rising in recent years (Abedizadeh et al. 2024). It is also among the most prevalent malignancies of the digestive tract worldwide. With the growing and aging population, it is estimated that by 2035, the number of deaths from colon and rectal cancers will increase by 60.0% and 71.5%, respectively, across all countries (Araghi et al. 2019). In recent years, shifts toward high‐fat, high‐calorie diets and a general decline in physical activity have contributed to an increasing trend in CRC incidence, with particularly pronounced increases observed in certain regions. Based on the 2025 edition of the Chinese Colorectal Cancer Diagnosis and Treatment Guidelines issued by the National Health Commission, a concise summary of its clinical manifestations and therapeutic strategies was provided (Department of Medical Administration, National Health Commission of the People's Republic of China, and Oncology Society of Chinese Medical Association 2025).

### 4.1 Clinical Manifestations of CRC

Clinically, CRC often manifests with insidious or nonspecific symptoms at the early stage, and patients frequently experience no overt discomfort, which contributes to delayed diagnosis. As tumor burden increases and disease progresses, characteristic manifestations gradually emerge, including abdominal pain, hematochezia, alterations in bowel habits, weight loss, and secondary anemia. In cases of locally advanced or progressive disease, continuous tumor invasion and expansion into the intestinal wall may lead to luminal narrowing or even complete obstruction; in severe cases, this can be complicated by intestinal obstruction, perforation, and cachexia, thereby markedly compromising patient prognosis (Madkhali et al. 2025). In advanced stages, persistent minor bleeding can lead to anemia. Chronic progressive anemia, malnutrition, and systemic toxicity symptoms caused by local ulceration and absorption of infectious toxins can result in weight loss, fatigue, general weakness, and even cachexia (Hossain et al. 2022). Acute perforation may trigger peritonitis. Hepatomegaly, ascites, and enlargement of the cervical and supraclavicular lymph nodes usually indicate late‐stage disease with tumor metastasis.

### 4.2 Therapeutic Approaches for CRC

With the rapid advances in medical technology, the treatment of CRC has evolved from traditional surgery and chemoradiotherapy to multimodal, precise, and individualized therapeutic system (Fadlallah et al. 2024). Surgical management has transitioned from traditional open resection to minimally invasive methods, including laparoscopic and robot‐assisted techniques, facilitating oncologic clearance with reduced surgical morbidity (Chen et al. 2025; Liu, Wu, et al. 2025). The optimal management of colorectal cancer requires an integrated assessment of tumor location, TNM stage, molecular profiling, and patient fitness, with personalized multimodal strategies developed within a multidisciplinary team framework (Kusumaningrum et al. 2024). While colon and rectal cancers share overarching systemic therapeutic principles, they diverge substantially in terms of their approaches to local control and the utilization of radiotherapy.

Surgical resection constitutes the cornerstone of CRC management. Radical colectomy is recommended whenever operability criteria are fulfilled. For tumors extending beyond the muscularis propria or with regional nodal involvement, adjuvant chemotherapy is indicated. Surgery likewise remains the principal modality for rectal cancer, with preoperative radiotherapy considered in cases of perirectal tissue invasion based on individualized clinical assessment. If postoperative pathology confirms deep muscular invasion or lymph node metastasis, adjuvant radiotherapy followed by systemic chemotherapy should be implemented. For patients presenting with hepatic metastases, surgical resection is strongly advocated when the metastatic lesions are deemed resectable. In cases where surgery is not feasible but the disease burden remains relatively confined, locoregional therapeutic strategies, such as transarterial chemoembolization, may be considered.

The choice of surgical approach is determined by tumor location, extent of invasion, its relationship to adjacent organs, and the patient's overall clinical condition. In early‐stage disease, curative outcomes can be achieved through en bloc resection of the primary tumor, the involved bowel segment, and regional lymph nodes. In advanced cases or those complicated by obstruction, perforation, or other sequelae, palliative surgery is generally performed to relieve intestinal obstruction, repair perforation, or mitigate symptoms. In recent years, resection of hepatic and pulmonary metastases has been shown to prolong survival and improve quality of life to a certain extent, with combined chemoradiotherapy further increasing therapeutic efficacy.

Chemotherapy constitutes a pivotal component of the multimodal management of colorectal cancer, primarily serving as adjuvant therapy for patients with Dukes' stage B and C disease, as well as a palliative treatment for those with advanced‐stage tumors. Neoadjuvant chemotherapy can reduce the preoperative tumor burden and decrease the incidence of occult micrometastases, thereby improving the likelihood of achieving R0 resection (Van Cutsem et al. 2016). Commonly employed regimens include CF (LV/5‐FU), FOLFIRI (CPT‐11/LV/5‐FU), CAPEOX (capecitabine/oxaliplatin), and FOLFOX (oxaliplatin/LV/5‐FU). Among these, oxaliplatin‐based regimens (FOLFOX and CAPEOX) and the irinotecan‐based regimen FOLFIRI are currently recognized as effective therapeutic standards (Biniaz et al. 2024; Kam et al. 2025; Smith et al. 2024). Radiotherapy is primarily indicated for rectal cancer patients. Preoperative radiotherapy can reduce tumor size, decrease lymph node metastasis, lower the risk of local recurrence, and improve sphincter preservation rates (Wang et al. 2025). Postoperative radiotherapy is indicated for patients with incomplete resection, adjacent tissue invasion, or inadequate lymph node dissection, with the goal of reducing the risk of recurrence and metastasis. Total neoadjuvant therapy (TNT) involves administering all adjuvant treatment prior to surgery. This approach not only improves patient treatment adherence but also maximally reduces tumor burden and eradicates potential micrometastases, thereby further increasing the likelihood of curative resection and improving long‐term survival outcomes (Bahadoer et al. 2021; Conroy et al. 2021).

Molecular targeted therapy constitutes a key approach in CRC management. Monoclonal antibodies, including cetuximab and bevacizumab, exhibit pronounced efficacy in advanced disease, although their high cost restricts widespread accessibility.

By integrating the expertise of surgery, medical oncology, radiology, radiotherapy, and pathology, comprehensive treatment strategies encompassing neoadjuvant therapy, surgical resection, and postoperative adjuvant therapy have been established, significantly improving curative outcomes and survival even in complex cases, including those with hepatic metastases (Filoni et al. 2023; Li, Chen, et al. 2020). Moreover, as TCM has demonstrated increasing effectiveness in tumor prevention and treatment, its unique theories and therapeutic approaches have gradually gained attention in the management of CRC. Within the theoretical framework of TCM in China, CRC can be differentiated into syndromes such as blood‐stasis toxin obstruction, damp‐heat accumulation, spleen‐kidney yang deficiency, qi and blood deficiency, and liver‐kidney yin deficiency, with treatments including herbal medicine, acupuncture, and integrative approaches combining TCM and Western medicine. The application of herbal medicine isbased primarily on its antitumor activity, immune modulation, mitigation of the side effects of radiotherapy and chemotherapy, and improvement in quality of life. CRC treatment of CRC often imposes a significant psychological burden on patients, leading to anxiety. Acupuncture can effectively alleviate stress and anxiety by promoting blood circulation and regulating nervous system function (Yang, Yang, et al. 2021). Integrative TCM and Western medicine approaches are considered optimal for CRC treatment, combining surgery with TCM interventions, herbal‐assisted chemotherapy, and radiotherapy to alleviate postoperative symptoms, reduce treatment‐related side effects, and improve therapeutic outcomes (Li et al. 2022). A comprehensive investigation into the theoretical foundations and clinical applications of TCM for CRC, along with Western medicine approaches and an integrative treatment model, provides innovative strategies for applying TCM and Western medicine combinations in clinical CRC management.

## 5 Botanical Characteristics, Pharmacognosy, Identification, and Quality Control of *Lonicerae Japonicae Flos*

**FIGURE 1:**
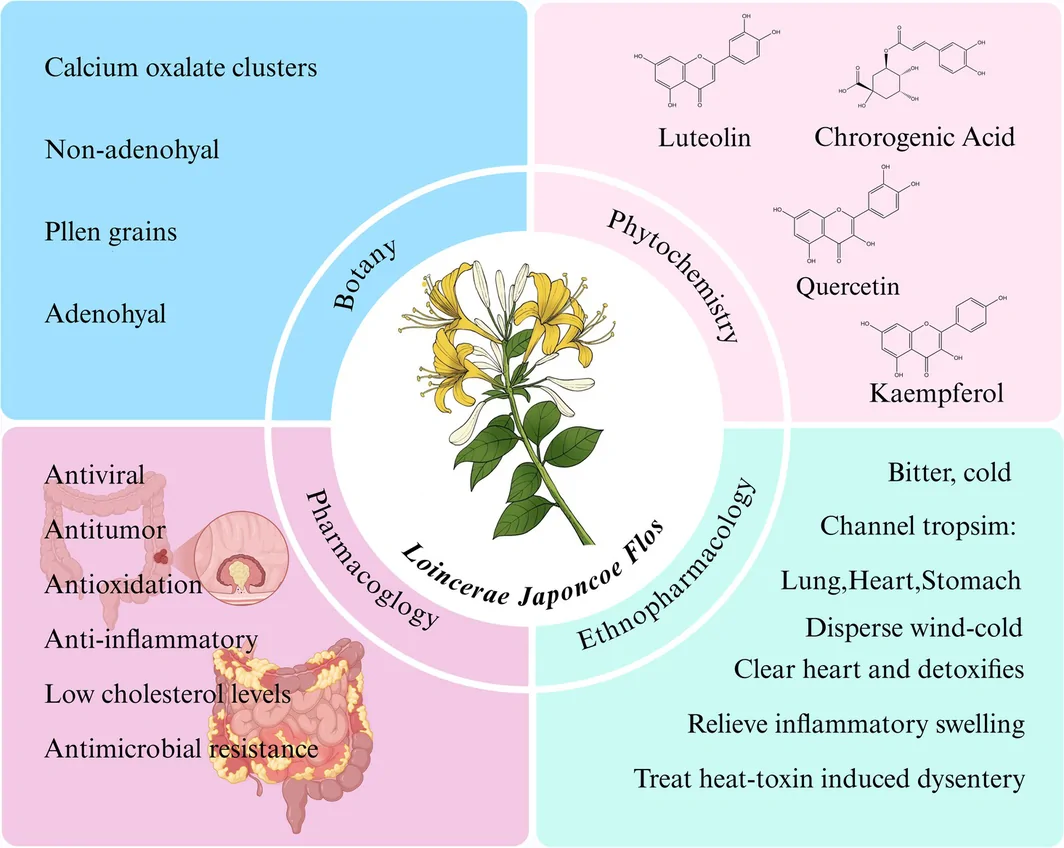
Integrated schematic overview of the botanical characteristics, major bioactive constituents, pharmacological activities, and ethnopharmacological properties of LJF, highlighting its multi‐target mechanisms and translational potential as a medicine–food homologous resource.

LJF, a widely used traditional Chinese medicinal herb, is the dried flower buds or flowers in the early blooming stage of *Lonicera japonica* Thunb. (Caprifoliaceae). The buds are harvested in early summer before the flowers fully bloom and then dried. The accepted scientific name of the species, *Lonicera japonica* Thunb., was verified using the World Flora Online database (http://www.worldfloraonline.org, accessed March 28, 2026). This nomenclature frequently appears in TCM prescriptions and academic research on herbal medicine. LJF has a sweet taste and cold nature, and it is associated with the lung, heart, and stomach meridians. In the medical field, it is highly regarded for its heat‐clearing, detoxifying, and wind‐heat dispersing properties (Zang et al. 2024). Clinically, it is commonly used to treat conditions such as abscesses, boils, pharyngalgia, erysipelas, febrile dysentery, and wind‐heat or cold‐heat syndromes. LJF exhibits remarkable broad‐spectrum antimicrobial activity and is therefore often metaphorically referred to as “natural antibiotic” of TCM, a designation intended only to highlight its antimicrobial properties (Li, Zhang, He, et al. 2024; Niu et al. 2026; Shang et al. 2011). It should be stored in a cool, dry place, protected from moisture and pests. It provides an integrated overview of the botanical characteristics, principal bioactive constituents, pharmacological activities, and ethnopharmacological properties of LJF, underscoring its multilevel synergistic value and significant translational potential as a medicine–food homologous resource in both modern pharmacological research and traditional application systems (Figure 1).

### 5.1 Growth Environment

LJF is highly adaptable, capable of tolerating both cold and high temperatures, and prefers sunny locations with well‐drained, loose soil. It thrives in moist environments but waterlogging must be avoided. LJF can also tolerate partial shade and poor soil conditions. Growing in well‐ventilated areas helps reduce the presence of pests and occurrence of diseases, making this species resilient to both dry and moist soil conditions and relatively undemanding regarding soil type. However, it grows optimally in sandy loam soils that are moist, fertile, and deep. LJF is widely distributed and commonly found in shrublands or sparse forests on hillsides, among rock piles, along roads at the foot of mountains, and at the edges of village fences (Li, Li, et al. 2020).

### 5.2 Characteristics, Identification and Quality Control

The morphological characteristics, methods to identify, and quality control of LJF were reviewed with reference to the relevant provisions of the 2025 edition of the Pharmacopeia of the People's Republic of China, Part I (Chinese Pharmacopeia Commission 2025).

#### 5.2.1 Morphological Characteristics

The flower buds of LJF are slender and clavate, with a narrower base (approximately 1.5 mm in diameter) gradually thickening toward the apex (approximately 3 mm in diameter), and a length of 2–3 cm. The buds are slightly curved overall. Fresh LJF appears yellowish‐white or light green, but prolonged storage leads to darkening because of oxidation or phenolic transformations. This color change can serve as an indicator of storage duration. The surface is covered with dense short pubescence, a distinguishing feature of the *Lonicera* genus, which is observable under a microscope. The base of the bud may retain 1–2 leaf‐like bracts, indicating that part of the peduncle structure was preserved during harvest. The calyx, located at the base, is green, and approximately 2 mm long, with five triangular lobes at the apex edged with hairs. At anthesis, the corolla is slender and tubular, terminating in a distinctly bilabiate limb. There are five stamens attached to the inner wall of the corolla tube, and a single pistil with an enlarged stigma and a smooth, glabrous ovary. This glabrous ovary helps distinguish LJF from *Lonicera confusa* ( *L. confusa* ), which has a hairy ovary. Due to the presence of volatile oils, LJF has a fresh, fragrant aroma. If the scent is weak or rancid, it may indicate improper storage or adulteration. Taste‐wise, it is initially slightly bitter, followed by a sweet aftertaste. Excessive bitterness or noticeable sourness may suggest contamination or molding. Meticulous differentiation is essential when dealing with commonly confounded species. For example, owing to their similar floral morphology, LJF is frequently confused with closely related species, such as *Lonicera macranthoides* Hand.‐Mazz., *Lonicera hypoglauca* Miq., *L. confusa* DC. and *Lonicera fulvotomentosa* Hsu et S.C.Cheng. Nevertheless, these species can be readily distinguished by differences in flower bud size, pubescence characteristics, and phytochemical composition. Additionally, the highly toxic *Gelsemium elegans* has occasionally been misidentified as honeysuckle during harvesting, underscoring the importance of accurate botanical identification.

#### 5.2.2 Identification

##### 5.2.2.1 Microscopic Identification

Microscopic examination revealed that the glandular trichomes of LJF are densely distributed, with apices exhibiting inverted conical, nearly spherical, or slightly flattened spherical shapes. Each trichome apex consists of 4–33 cells arranged in 2–4 discernible layers, with diameters ranging from 30 to 108 μm. The stalks are composed of 1–5 longitudinally aligned cells that extend up to 700 μm in length with thin cell walls and transparent cytoplasm. Two types of nonglandular trichomes are observed: (1) thick‐walled, unicellular hairs reaching up to 900 μm in length, exhibiting markedly thickened walls and surfaces adorned with fine wart‐like or vesicular projections, occasionally displaying spiral striations; (2) thin‐walled, unicellular hairs that are extremely elongated, often curved or contracted in shape, with surfaces covered by minute wart‐like protuberances and thin, transparent cell walls. Calcium oxalate druse crystals are ubiquitous, with diameters ranging from 6 to 45 μm. Pollen grains are subglobular or triangular, characterized by dense short spines and fine granular ornamentation on the exine, and exhibit tricolpate morphology. These micromorphological features are typically employed for the morphological identification of LJF.

##### 5.2.2.2 Chemical Identification

First, 0.2 g of LJF powder was weighed, and 5 mL of the polar solvent methanol was added. The mixture was allowed to stand for 12 h and then filtered; the filtrate served as the test solution. Moreover, a reference solution of the CGA standard was prepared and dissolved in the same solvent (methanol) at a concentration of 1 mg/mL to ensure comparability with the same detection system. As a characteristic component of LJF, CGA exhibits specific chromatographic behavior.

Silica gel H TLC plates were used as the stationary phase, and the upper organic phase of butyl acetate–formic acid–water (7:2.5:2.5) was selected as the developing solvent. This ternary system allows effective separation of the target components by adjusting the polarity. Spot volumes were 10–20 μL for the test solution and 10 μL for the reference, with differential spotting increasing the detection sensitivity of minor components.

In accordance with the TLC method (General Rule 0502, Chinese Pharmacopeia), 10–20 μL of the test solution and 10 μL of the reference solution were spotted on the same silica gel H plate and developed with the upper organic phase (butyl acetate–formic acid–water (7:2.5:2.5)). After development, the plate was air‐dried and observed under UV light at 365 nm for fluorescence characterization. Upon dissociation in the acidic developing solvent, the conjugated structures emit blue or blue–white florescence under UV excitation. Positive identification requires that the test solution exhibits fluorescence spots that completely overlap with the reference solution in the Rf range of 0.3–0.5 (with the exact value calibrated according to temperature and humidity), with a regular shape and clear edges.

This method complies with Chinese Pharmacopeia General Rule 0502, with blank controls used to eliminate matrix interference. System suitability tests must ensure solvent front migration of ≥ 8 cm and a spot resolution > 1.5. When fluorescent spots with matching Rf values appeared in the test solution, the presence of LJF was specifically confirmed, allowing differentiation from closely related species such as *L. confusa* , the content of which is significantly lower or which contains interfering components.

#### 5.2.3 Quality Control

LJF quality is assessed mainly by quantifying its key bioactive constituents, with phenolic acids and flavonoids serving as critical markers of intrinsic quality and pharmacological stability (Zeng et al. 2024). Currently, high‐performance liquid chromatography (HPLC) is widely employed for the identification and quality assessment of traditional Chinese medicines due to its high sensitivity, excellent reproducibility, and superior separation capability. It also serves as the standard method for LJF quality control as stipulated in the Pharmacopeia of the People's Republic of China (Niu et al. 2012; Zhang, Yu, Yang, et al. 2024).

Phenolic acids are among the most representative chemical constituents of LJF and serve as the core quality markers recognized in the current Pharmacopeia. Using CGA, 3,5‐di‐O‐Caffeoylquinic acid, and 4,5‐di‐O‐Caffeoylquinic acid as key analytes, the Pharmacopeia mandates a minimum of 1.5% CGA and a total phenolic acid content of 3.8% (dry weight) to ensure the fundamental quality of LJF (He et al. 2019). In addition to phenolic acids, flavonoids are integral to the quality of LJF. Luteoloside, a characteristic flavonoid, is employed as an auxiliary marker, with a mandated minimum content of 0.050% (dry weight), enhancing the multi‐parameter quality evaluation framework.

An integrated approach combining ultra‐fast liquid chromatography–triple quadrupole mass spectrometry (UFLC‐QTRAP‐MS/MS) with multivariate statistical analysis can also be employed for quality assessment and to reveal differential constituents in LJF (Cai et al. 2021). In addition, methods such as “one assay–multiple evaluations” and the use of traditional Chinese medicine quality markers are widely applied for herbal quality control.

The simultaneous quantification of phenolic acids and flavonoids provides a comprehensive reflection of LJF quality, controllability, and stability, thereby offering a reliable basis for pharmacological research and rational clinical application.

## 6 Investigation of the Bioactive Constituents of LJF for the Prevention and Treatment of UC and CRC

The effective chemical constituents of LJF that alleviate UC are diverse and form the core of its pharmacological activity. Among them, volatile oils such as linalool and geraniol exhibit significant antibacterial and anti‐inflammatory activities and play a positive role in maintaining immune system balance (Singh and Mishra 2024). Flavonoids, such as Lut, exhibit anti‐inflammatory, antitumor, and antioxidant properties, along with the capacity to modulate inflammatory responses (Lv et al. 2021; Özerkan 2023). Organic acids primarily exert their effects through immune regulation in synergy with antibacterial activity (Harwansh et al. 2024). Triterpenoid saponins can bidirectionally regulate immune responses and increase the stability of the intestinal mucosa, whereas polysaccharides contribute to modulating the gut microbiota and strengthening barrier defense functions (Li, Zhang, He, et al. 2024; Ma et al. 2024).

### 6.1 Volatile Oils

LJF contains a complex mixture of volatile oils, including primarily terpenoids (such as monoterpenes and sesquiterpenes) and aromatic compounds. The following are the volatile oil components in LJF (Table 1). These oils are typically colorless or pale yellow liquids, possess a distinct aromatic odor, are soluble in organic solvents but insoluble in water, and are sensitive to light and heat. Various extraction and analytical methods are employed for their analysis, among which steam distillation and gas chromatography–mass spectrometry (GC–MS) are commonly used. For example, linalool, a monocyclic terpene with a hydroxyl group and a carbon–carbon double bond in its chemical structure. This structural arrangement endows it with distinct physicochemical properties. The presence of a hydroxyl group increases the polarity of the molecule, making it somewhat soluble in hydrophilic solvents, whereas the presence of a carbon–carbon double bond provides a reactive site for chemical reactions, forming the structural basis for its antibacterial and anti‐inflammatory activities (Gavit et al. 2024). Eugenol is a phenylpropanoid derivative that possesses a basic phenylpropane skeleton. The hydroxyl and ether groups attached to this backbone influence the distribution of the molecule's electron density, thereby altering its interaction patterns with biomolecules. When in contact with the phospholipid bilayer of bacterial cell membranes, eugenol can disrupt the lipid packing order, thereby exerting antibacterial effects (Qu et al. 2024).

**TABLE 1:** The volatile oil components in LJF.

Molecule name	CAS	MF	PubChem CID	References
Germacrene D	23986‐74‐5	C_15_H_24_	5317570	Li, Zhang, He, et al. (2024)
Benzaldehyde	100‐52‐7	C_7_H_6_O	240	Cai et al. (2013)
Hexanal	66‐25‐1	C_6_H_12_O	6184	Cai et al. (2013)
Farnesal	19317‐11‐4	C_15_H_24_O	68150	Li, Zhang, He, et al. (2024)
Phenethyl alcohol	60‐12‐8	C_8_H_10_O	6054	Wu et al. (2024)
Linalool	78‐70‐6	C_10_H_18_O	6549	Li, Zhang, He, et al. (2024)
Eucalyptol	470‐82‐6	C_10_H_18_O	2758	Zhang, Sun, et al. (2023)
Geraniol	106‐24‐1	C_10_H_18_O	637566	Cai et al. (2013); Li, Zhang, He, et al. (2024)
Eugenol	97‐53‐0	C_10_H_12_O_2_	3314	Qu et al. (2024)
Copaene	3856‐25‐5	C_15_H_24_	12303902	Chen, Zou, et al. (2024)
Linalyl Oxide	60047‐17‐8	C_10_H_18_O_2_	22310	Wang et al. (2016)
Carvacrol	499‐75‐2	C_10_H_14_O	10364	Ma et al. (2024)
cis‐3‐Hexen‐1‐ol	928‐96‐1	C_6_H_12_O	5281167	Wu et al. (2024)
(−)‐alpha‐Pinene	7785‐26‐4	C_10_H_16_	440968	Li, Zhang, He, et al. (2024)
Alpha‐Terpineol	98‐55‐5	C_10_H_18_O	17100	Li, Zhang, He, et al. (2024)
Farnesol	4602‐84‐0	C_15_H_26_O	445070	Li, Zhang, He, et al. (2024)
Nerolidol	7212‐44‐4	C_15_H_26_O	5284507	Chen, Zou, et al. (2024); Li, Zhang, He, et al. (2024)
Nerol	106‐25‐2	C_10_H_18_O	643820	Chen, Zou, et al. (2024)
Citronellol	106‐22‐9	C_10_H_20_O	8842	Chen, Zou, et al. (2024)
Geranyl acetate	105‐87‐3	C_12_H_20_O_2_	1549026	Chen, Zou, et al. (2024)
(+)‐alpha‐Cadinene	24406‐05‐1	C_15_H_24_	12306048	Li, Zhang, He, et al. (2024); Wang et al. (2012)
(+)‐delta‐Cadinene	483‐76‐1	C_15_H_24_	441005	Li, Zhang, He, et al. (2024); Wang et al. (2012)

The volatile oil of LJF exhibits antibacterial and anti‐inflammatory effects, with its anti‐inflammatory mechanism potentially related to the regulation of inflammatory mediator release and the inhibition of inflammatory cell activation. In the treatment of UC, the volatile oil may serve as an adjunct therapy by alleviating intestinal inflammation and improving the microecological environment in the gut (Li, Zhang, Luan, et al. 2024).

### 6.2 Flavonoid Compounds

Luteoloside (Lut) is a flavonoid glycoside present in LJF that is composed of Lut and glucose. Lut has the characteristic C6‐C3‐C6 backbone structure of flavonoid (Zhu, Sun, et al. 2024). The phenolic hydroxyl groups in its structure confer strong antioxidant activity by enabling the donation of hydrogen atoms to neutralize free radicals and thereby interrupt free radical chain reactions (Huang et al. 2023). The following are the flavonoid bioactive components present in LJF (Table 2). In addition to Lut, LJF contains various other flavonoid compounds, such as Que. and Kae (Gangwar et al. 2025). These flavonoids also contribute to alleviating the symptoms of UC. Que. is a widely distributed flavonoid in plants that has multiple bioactivities, including anti‐inflammatory, antiviral, antioxidant, and immunomodulatory effects (Chiang et al. 2023; Manjunath and Thimmulappa 2022; Zhou et al. 2023; Zhu, Han, et al. 2024).

**TABLE 2:** Flavonoid components in LJF.

Molecule name	CAS	MF	PubChem CID	References
Kaempferol	520‐18‐3	C_15_H_10_O_6_	5280863	Ni et al. (2015)
Luteoloside	5373‐11‐5	C_21_H_20_O_11_	5280637	Zhang, Yu, Sun, et al. (2024)
Rutin	153‐18‐4	C_27_H_30_O_16_	5280805	Ge et al. (2022)
Hyperoside	482‐36‐0	C_21_H_20_O_12_	5281643	Wang et al. (2016)
Isoquercitrin	21637‐25‐2	C_21_H_20_O_12_	5484006	Zhang, Yu, Sun, et al. (2024)
Chrysoeriol	491‐71‐4	C_16_H_12_O_6_	5280666	Chen, Zou, et al. (2024)
Luteolin	491‐70‐3	C_15_H_10_O_6_	5280445	Cai et al. (2021); Wang et al. (2016)
Quercetin	117‐39‐5	C_15_H_10_O_7_	5280343	Ge et al. (2022)
Apigenin	520‐36‐5	C_15_H_10_O_5_	5280443	Wang et al. (2016)
Lonicerin	25694‐72‐8	C_27_H_30_O_15_	5282152	Wang et al. (2016)

### 6.3 Organic Acids

CGA is widely recognized as the principal bioactive constituent of LJF and the most abundant phenolic acid present in this herb. Also referred to as caffeoylquinic acid, CGA is chemically defined as 3‐O‐caffeoylquinic acid, a condensed phenolic acid formed through an ester linkage between caffeic acid and quinic acid. Biosynthetically, it belongs to the phenylpropanoid family and is generated via the shikimate pathway during aerobic metabolism in plants. Structurally, CGA contains ester bonds, conjugated double bonds, polyphenolic moieties, and ortho‐dihydroxyl groups, all of which collectively underpin its broad spectrum of biological activities. As an organic acid bearing both phenolic hydroxyl and carboxyl functional groups, CGA is amphiphilic and exhibits hydrophilic properties with a certain degree of lipophilicity. This physicochemical versatility enables it to exert biological effects across diverse physiological microenvironments. Notably, the phenolic hydroxyl groups can serve as electron donors to scavenge free radicals during antioxidative processes, whereas the carboxyl group may participate in acid–base interactions with target proteins, thereby facilitating molecular recognition and bioactivity. The organic acid constituents of LJF were extracted from the aforementioned databases after database screening and manual verification (Table 3). Accumulating evidence indicates that CGA possesses a wide array of pharmacological activities, including antioxidant (Chen, Zhou, et al. 2024), anti‐inflammatory (Jiao et al. 2024), antibacterial (Chen et al. 2022), antiviral (Abaidullah et al. 2021), antitumor (Wang, Du, and Chen 2020), hypolipidemic (Meng et al. 2024), hypoglycemic (Qin et al. 2021), and immunomodulatory effects (Zhu, Shen, et al. 2024). Owing to this multifunctional bioactivity profile, CGA has attracted considerable interest and has demonstrated substantial application potential in the food, pharmaceutical, and chemical industries.

**TABLE 3:** The organic acid components in LJF.

Molecule name	CAS	MF	Pubchem CID	References
Chlorogenic acid	327‐97‐9	C_16_H_18_O_9_	1794427	Chen, Zou, et al. (2024)
Isochlorogenic acid A	2450‐53‐5	C_25_H_24_O_12_	6474310	Chen, Zou, et al. (2024)
Isochlorogenic acid B	14534‐61‐3	C_25_H_24_O_12_	5281780	Chen, Zou, et al. (2024)
Isochlorogenic acid C	57378‐72‐0	C_25_H_24_O_12_	6474309	Chen, Zou, et al. (2024)
Isochlorogenic acid	534‐61‐2	C_16_H_18_O_9_	6436237	Chen, Zou, et al. (2024)
Neochlorogenic acid	906‐33‐2	C_16_H_18_O_9_	5280633	Wang et al. (2016)
Cryptochlorogenic acid	905‐99‐7	C_16_H_18_O_9_	9798666	Wang et al. (2016)
Caffeic acid	331‐39‐5	C_9_H_8_O_4_	689043	Chen, Zou, et al. (2024)
Protocatechuic acid	99‐50‐3	C_7_H_6_O_4_	72	Chen, Zou, et al. (2024)
Astragalin	480‐10‐4	C_21_H_20_O_11_	5282102	Zhang, Yu, Sun, et al. (2024)
Rhoifolin	17306‐46‐6	C_27_H_30_O_14_	5282150	Wang et al. (2016)
3,4,5‐Tricaffeoylquinic acid	86632‐03‐3	C_34_H_30_O_15_	6440783	Chen, Zou, et al. (2024)

### 6.4 Triterpenoid Saponins

LJF contains some triterpenoid saponins, such as Loniceroside A and Loniceroside B. These saponins also play a role in alleviating UC symptoms. The following triterpenoid saponins constituents of LJF were identified through database screening and manual verification (Table 4). Structurally, triterpenoid saponins are composed of triterpene aglycone and sugar moieties, with the aglycone exhibiting either a tetracyclic or pentacyclic triterpene framework, including oleanane‐type and ursane‐type scaffolds. These intricate structures confer diverse spatial conformations and physicochemical properties to triterpenoid saponins, whereas variations in the length, branching, and linkage of the sugar chains further increase their structural diversity (Chen, Zou, et al. 2024). Triterpenoid saponins exhibit pronounced anti‐inflammatory and antitumor activities, which are likely mediated through the modulation of multiple disease‐related signaling pathways.

**TABLE 4:** Triterpenoid saponins components in LJF.

Molecule name	CAS	MF	PubChem CID	References
Cauloside C	20853‐58‐1	C_41_H_66_O_13_	13878151	Li, Zhang, Yu, et al. (2024)
Loniceroside A	155740‐20‐8	C_52_H_84_O_21_	10102583	Li, Zhang, Yu, et al. (2024)
Loniceroside B	155740‐21‐9	C_58_H_94_O_25_	10057143	Li, Zhang, Yu, et al. (2024)

### 6.5 Polysaccharides

LJF polysaccharides (LJP) are considered the principal bioactive constituents of LJF. LJP are high‐molecular‐weight carbohydrates that consist of monosaccharides such as glucose, galactose, and mannose linked through glycosidic bonds. Their molecular chains exhibit varying degrees of branching, endowing them with notable water solubility and viscosity (Yang, Yu, et al. 2023). The structural complexity of LJP underpins its diverse biological activities. Polysaccharides obtained via different extraction methods vary in molecular weight, monosaccharide composition, and glycosidic linkage type, which in turn influence their pharmacological efficacy (Zhang et al. 2020). Liu et al. (2024) reported a strong relationship between the structural characteristics of LJP and its anti‐inflammatory potency. Differences in molecular weight, monosaccharide composition, and glycosidic linkage types among various polysaccharides markedly affect their anti‐inflammatory efficacy.

Polysaccharides can modulate the immune system in UC mice, suppressing excessive immune responses and alleviating intestinal inflammation. Additionally, they enhance the body's antioxidant capacity, mitigating oxidative stress–induced damage to intestinal tissues. Moreover, polysaccharides may reshape the gut microbiota, promoting the proliferation of beneficial bacteria and improving the intestinal microecological environment, thereby potentially exerting therapeutic effects against UC.

### 6.6 Other Bioactive Components

LJF also contains additional bioactive constituents, notably iridoids, a class of monoterpenoids characterized by a cyclopentane–pyran core structure, which exhibit diverse pharmacological activities, including antimicrobial, neuroprotective, antitumor, hepatoprotective, and antidepressant effects (Elosaily et al. 2026; Guo et al. 2025; Wang, Gong, et al. 2020; Xue et al. 2025; Table 5).

**TABLE 5:** Iridoid components in LJF.

Molecule name	CAS	MF	PubChem CID	References
(Z)‐Aldosecologanin	82474‐97‐3	C_34_H_46_O_19_	6440698	Zhang, Yu, Sun, et al. (2024)
Sweroside	14215‐86‐2	C_16_H_22_O_9_	161036	Li, Zhang, He, et al. (2024); Song et al. (2006)
Secologanin	19351‐63‐4	C_17_H_24_O_10_	161276	Wang et al. (2013)
Loganin	18524‐94‐2	C_17_H_26_O_10_	87691	Zhang et al. (2022)
(E)‐Aldosecologanin	471271‐55‐3	C_34_H_46_O_19_	45783101	Zhang, Yu, Sun, et al. (2024)
Loganic acid	22255‐40‐9	C_16_H_24_O_10_	89640	Zhang et al. (2022)
Morroniside	25406‐64‐8	C_17_H_26_O_11_	11228693	Zhang, Yu, Sun, et al. (2024)

## 7 Molecular Mechanisms Underlying the Amelioration of UC by the Bioactive Constituents of LJF

**FIGURE 2:**
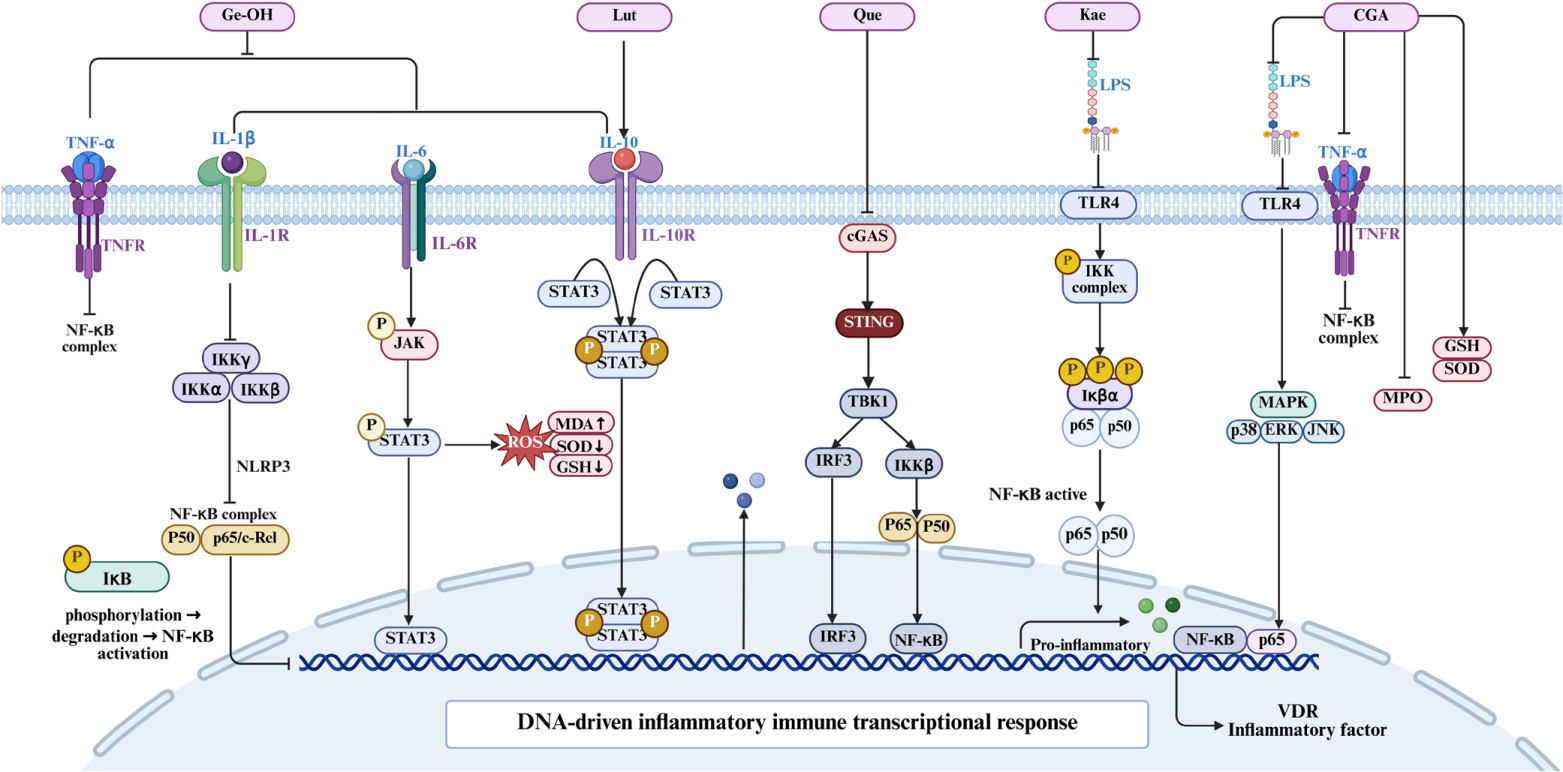
Molecular mechanisms underlying the anti‐UC effects of bioactive constituents from LJF. Bioactive constituents of LJF alleviate colonic inflammation by modulating NF‐κB, NLRP3, and MAPK signaling, reducing pro‐inflammatory cytokines, and exerting antioxidant effects. CGA, chlorogenic acid; cGAS, Cyclic GMP‐AMP synthase; ERK, extracellular signal‐regulated kinase; Ge‐OH, geraniol; GSH, glutathione; IKK, IκB kinase; IKKα, IκB kinase α; IKKβ, IκB kinase β; IKKγ, IκB kinase γ; IL‐10, interleukin‐10; IL‐10R, interleukin‐10 receptor; IL‐1R, interleukin‐1 receptor; IL‐1β, interleukin‐1β; IL‐6, interleukin‐6; IL‐6R, interleukin‐6 receptor; IRF3, interferon regulatory factor 3; IκB, Inhibitor of NF‐κB; JAK, janus kinase; JNK, c‐Jun N‐terminal kinase; Kae, kaempferol; LPS, lipopolysaccharide; Lut, luteolin; MAPK, mitogen‐activated protein kinase; MDA, malondialdehyde; MPO, myeloperoxidase; NF‐κB, nuclear factor kappa B; NLRP3, NOD‐like receptor family pyrin domain containing 3; p38, p38 mitogen‐activated protein kinase; Que, quercetin; ROS, reactive oxygen species; SOD, superoxide dismutase; STAT3, signal transducer and activator of transcription 3; STING, stimulator of interferon genes; TBK1, TANK‐binding kinase 1; TLR4, toll‐like receptor 4; TNFR, tumor necrosis factor receptor; TNF‐α, tumor necrosis factor‐α; VDR, Vitamin D receptor.

Despite the marked differences in chemical structures among the various bioactive constituents of LJF, these compounds exhibit highly similar mechanisms in the amelioration of UC, primarily the regulation of inflammatory responses, attenuation of oxidative stress, preservation of intestinal barrier integrity, remodeling of the gut microbiota, and maintenance of immune homeostasis (Figure 2).

### 7.1 Suppression of Inflammatory Responses and Pro‐Inflammatory Cytokines

The volatile oil components of LJF exhibit significant anti‐inflammatory activity, primarily through the modulation of inflammation‐related cells and signaling pathways (Stojanović et al. 2024). Studies have shown that oral geraniol (Ge‐OH) can improve acute experimental colitis in mice by inhibiting proinflammatory cytokines, especially tumor necrosis factor‐alpha (TNF‐α), interleukin‐1 beta (IL‐1β), and interleukin‐6 (IL‐6), and modulating nuclear factor kappa‐B (NF‐κB) signaling (Medicherla et al. 2015). Yang, Duan, Yan, et al. (2025) demonstrated that appropriate doses of Lut significantly mitigated clinical manifestations in UC mice, including body‐weight loss, colon shortening, and histological injury, while robustly reducing proinflammatory cytokine levels in both serum and colonic tissues. Furthermore, Lut markedly increased the expression of the anti‐inflammatory cytokine interleukin‐10 (IL‐10), thereby effectively restoring the colonic barrier integrity disrupted by dextran sulfate sodium (DSS). Similarly, Xue et al. (2023) reported that Lut ameliorated DSS‐induced colitis by improving colonic inflammation, reinforcing intestinal barrier function, and limiting the production of proinflammatory cytokines in colonic tissues. Mechanistically, these effects were associated with the inhibition of inhibitor of κB kinase α/β (IKKα/β), subsequent suppression of NF‐κB signaling, and the blockade of macrophage activation and chemotactic migration, underscoring the therapeutic potential of Lut in DSS‐induced colitis. Preclinical studies suggest that CGA may exert anti‐inflammatory effects by inhibiting the activation of the NF‐κB and mitogen‐activated protein kinase (MAPK) signaling pathways, which may contribute to reduced production of pro‐inflammatory mediators (Zhao et al. 2023). Substantial evidence indicates that CGA exerts pronounced anti‐inflammatory effects and plays a crucial role in alleviating the manifestations of ulcerative colitis. NF‐κB is a pivotal transcription factor in the pathogenesis of ulcerative colitis, as diverse stimuli can trigger its activation, thereby initiating the transcription and expression of a broad spectrum of inflammation‐related genes. Notably, studies in interleukin‐10‐knockout mice have demonstrated that CGA markedly reduces the elevated expression of inducible nitric oxide synthase, IL‐1β, and TNF‐α, corroborating its anti‐inflammatory efficacy in colitis‐associated conditions (Lee et al. 2020). In parallel, CGA has been shown to mitigate DSS‐induced colonic mucosal injury by modulating the MAPK/ERK/JNK signaling cascade, leading to reduced production of inflammatory mediators, attenuation of oxidative stress, and suppression of apoptosis. These protective effects are accompanied by decreased protein expression of ERK1/2, p‐ERK, p38, p‐p38, JNK, and p‐JNK (Gao et al. 2019). Triterpenoid saponins exhibit notable anti‐inflammatory properties, which are likely mediated through the modulation of inflammatory signaling pathways.

### 7.2 Mitigation of Oxidative Stress

Oxidative stress plays a pivotal role in the pathogenesis of UC (Tratenšek et al. 2024). In mammalian cells, oxygen metabolism constitutes a fundamental process essential for sustaining vital cellular functions and inevitably gives rise to a certain amount of reactive oxygen species (ROS) (Sun et al. 2024). Lut has antioxidant effects. It can increase the body's antioxidant capacity and reduce the production of oxidative stress products (Li, Guo, et al. 2023). Studies have shown that Lut increases the activity of antioxidant enzymes such as superoxide dismutase (SOD) and glutathione peroxidase (GSH‐Px) in the colonic tissue of UC mice, while decreasing malondialdehyde (MDA) levels. This reduction in oxidative stress mitigates damage to the intestinal mucosa and thereby protects intestinal tissue (Sahoo et al. 2023). CGA exhibits robust antioxidant capacity, effectively scavenging excessive ROS and thereby alleviating oxidative damage to the intestinal mucosa. More specifically, CGA increases the activities of key antioxidant enzymes, including SOD and GSH‐Px, while simultaneously reducing MDA levels. Through these coordinated actions, CGA helps preserve redox homeostasis within the intestinal milieu, enabling mucosal epithelial cells to withstand oxidative insults and thereby contributing to the maintenance of intestinal barrier integrity (Hu et al. 2024).

### 7.3 Restoration of Intestinal Mucosal Barrier Function and Preservation of Tight Junction Integrity

The intestinal barrier comprises mechanical, chemical, biological, and immune components, and its integrity is essential for maintaining intestinal homeostasis. Li, Zhao, et al. (2021) revealed that Lut treatment substantially improved epithelial barrier function, an effect that may be mediated through the attenuation of SHP‐1‐dependent signaling and the downstream signal transducer and activator of transcription 3 (STAT3) pathway, ultimately protecting the intestinal epithelial barrier. Lut has been shown to restore the balance between NCR^−^‐ILC3s/NCR^+^‐ILC3s, promoting repair of the damaged intestinal epithelium and thereby mitigating UC (Xie et al. 2022). Experimental studies suggest that Que. may improve the integrity of tight junctions (TJs) in an aryl hydrocarbon receptor‐dependent manner, repairing intestinal barrier dysfunction and alleviating symptoms in UC mice (Wang, Xie, et al. 2024). Overall, Que. mitigated DSS‐induced colitis by reducing inflammation, enhancing tight junctions, and maintaining intestinal mucosal barrier stability. Kae is another anti‐inflammatory and antioxidant flavonoid in LJF. Kae has been shown to increase the expression of zonula occludens‐1 (ZO‐1), occludin, and claudin‐1 in mouse models of colitis, potentially repairing the intestinal mucosal barrier. Kae alleviates colitis by restoring the gut microbiota and suppressing the LPS‐TLR4‐NF‐κB signaling pathway (Qu et al. 2021).

### 7.4 Immune Cell Modulation and Homeostatic Regulation

Immune dysregulation constitutes a key mechanism underlying the persistent and relapsing nature of UC. The bioactive constituents of LJF may exert immunomodulatory effects by regulating immune cell functions and the expression of immune mediators. Yang et al. (Yang, Duan, Zeng, et al. 2025; Zhou et al. 2021) highlighted that Lut markedly alleviates DSS‐induced UC in mice through an AMP‐activated protein kinase (AMPK)/peroxisome proliferator–activated receptor γ (PPARγ)–dependent signaling cascade, effectively suppressing M1 macrophage polarization while promoting polarization toward the M2 phenotype. These findings underscore Lut as a promising naturally derived anti‐inflammatory agent for UC therapy, emphasizing its dual immunomodulatory and anti‐inflammatory potential. Gao et al. demonstrated that Que. inhibited the cGAS–STING pathway in intestinal macrophages in a DSS‐induced mouse model of UC, reduced M1 macrophage polarization, promoted M2 macrophage polarization, and improved intestinal barrier function, suggesting a potential protective role in UC (Gao et al. 2024). Que. can directly downregulate the protein expression of CXCL8, CXCR1, CXCR2, IL‐17A, and NF‐κB, further enhancing its protective effects against UC (Jiang et al. 2024). In terms of immunomodulation, researchers have reported that CGA modulates immune responses in murine macrophages and lymphocytes, including affecting T lymphocyte subsets, providing a broader experimental foundation and supporting evidence for its role in immune regulation (Guo et al. 2021). CGA can alter the proportions of T lymphocyte subsets, and in a mouse model of UC, intervention with CGA restored the Th1/Th2 imbalance, suppressed the overactivation of Th1 cells and thereby reduced cell‐mediated intestinal inflammation. Furthermore, CGA enhances the tolerogenic properties of dendritic cells, reduces their antigen‐presenting capacity, and mitigates excessive immune responses triggered by hyperactivated T lymphocytes (Zhang, Liu, et al. 2023). LJP confers a protective effect on splenic lymphocytes by mitigating immune cell apoptosis and restraining excessive immune responses, thereby exerting immunoprotective effects in DSS‐induced murine models of UC (Zhou et al. 2021).

### 7.5 Modulation of Gut Microbiota Homeostasis and Metabolic Function

Dysbiosis of the gut microbiota is a critical factor contributing to the onset of ulcerative colitis. Numerous bioactive constituents in LJF demonstrate significant modulatory effects on the gut microbiota. Within the intestinal inflammatory microenvironment, the volatile oils can specifically target pathogenic gut bacteria such as *Escherichia coli* and *Salmonella* (Cheng et al. 2023). By binding to specific targets on bacterial cell membranes, the volatile oils alter membrane permeability, leading to ionic imbalance and metabolic disruption within bacterial cells, ultimately inhibiting bacterial growth and reproduction, thereby reducing sources of inflammatory stimulation. Li, Du, et al. (2021) reported that Lut administration significantly increased the intestinal abundance of beneficial commensals, including *Roseburia* and butyrate‐producing bacteria. *Roseburia* species contribute to the amelioration of UC by modulating T‐lymphocyte differentiation and increasing the production of anti‐inflammatory cytokines, whereas butyrate‐producing bacteria generate butyrate, which has well‐established anti‐inflammatory properties and plays a critical role in maintaining intestinal barrier integrity. The enrichment of these taxa establishes a favorable feedback loop that dampens inflammatory responses and further facilitates UC remission. CGA exhibits antibacterial activity that suppresses the growth of harmful gut bacteria, thereby alleviating intestinal inflammation and facilitating UC remission. With respect to the modulation of the gut microbiota, CGA also exerts notable effects. Studies have revealed that CGA can modulate the composition and abundance of the gut microbiota in UC mice. Specifically, it promotes the proliferation of beneficial bacteria such as *Bifidobacterium* and *Lactobacillus* but suppresses the proliferation of harmful species such as *Escherichia coli* and *Enterococcus*. This rebalancing of the intestinal microecology helps alleviate gut inflammation. Zhang et al. (2019) demonstrated that CGA suppresses the release of proinflammatory cytokines, including MPO and TNF‐α, in the context of UC. Additionally, CGA mitigated the detrimental effects of DSS on gut microbial diversity, increasing overall microbial richness and the relative abundance of beneficial genera such as *Akkermansia* and *Lactobacillus*. An growing body of evidence indicates that the anti‐UC effects of triterpenoid saponins are closely linked to the gut microbiota. In the regulation of gut microecology, polysaccharides act as prebiotics, providing a nutritional substrate for beneficial bacteria such as *Bifidobacterium* and *Lactobacillus*, thereby promoting their proliferation. Concurrently, these species inhibit the growth of harmful bacteria, including *Escherichia coli* and *Clostridioides difficile*, ultimately improving the gut microbial composition and maintaining the microecological balance. Metabolites produced by beneficial bacteria, such as short‐chain fatty acids, can improve intestinal mucosal barrier function and attenuate inflammation. Zhou et al. (2021) demonstrated that high‐dose LJP (150 mg/kg) improved the gut microbial composition in DSS‐induced colitis mice by increasing the abundance of probiotics such as *Bifidobacterium* and *Lactobacillus*, while suppressing the abundance of pathogenic bacteria including *Escherichia coli* and *Enterococcus*. LJP further maintains microbial homeostasis by promoting the secretion of SIgA, thereby improving the intestinal microecological environment and alleviating inflammation. Polysaccharides have been shown to upregulate the expression of tight junction proteins in intestinal epithelial cells, including Occludin and ZO‐1, thereby enhancing intercellular cohesion and preventing the translocation of bacteria and endotoxins across the gut wall. Besides, polysaccharides can stimulate the secretion of mucins within the intestinal mucus layer, reinforcing a more robust physical barrier that safeguards the mucosa from damage and alleviates UC symptoms (Li, Lin, et al. 2023). Polysaccharides can orchestrate the gut microbiota composition and reinforce intestinal barrier integrity through specific signaling cascades, ultimately alleviating the manifestations of UC (Yang, Zhang, et al. 2023; Zhou et al. 2025).

### 7.6 Additional Mechanistic Insights

Lut ameliorates colitis in mice by suppressing inflammation, apoptosis, and autophagy via ERK‐mediated pathways, processes closely associated with the progression of UC toward CAC (Vukelić et al. 2020).

The multifaceted chemical constituents of LJF play critical and complementary roles in the management of UC through both discrete and synergistic mechanisms. Components such as volatile oils, flavonoids, organic acids, triterpenoid saponins, and polysaccharides combat UC from multiple aspects, including antimicrobial and anti‐inflammatory effects, antioxidant activity, immunomodulation, intestinal mucosal protection, and regulation of the gut microbiota. These bioactive compounds may mediate the potential protective effects through anti‐inflammatory and antioxidant pathways, modulating the gut microbiota composition, maintaining and protecting the integrity of the mucosal barrier, and regulating immunity. These findings provide mechanistic insights into the potential health benefits and future applications of LJF. Looking forward, these insights hold promise for the advancement of LJF‐derived precision therapeutics in the targeted management of UC. Nevertheless, current research still faces multiple challenges, including the incomplete elucidation of specific targets for certain constituents, the need for deeper exploration of synergistic interactions among components, and the refinement of quality control standards. Future investigations should leverage multidisciplinary approaches, such as systems biology and structural biology, to precisely delineate the mechanisms of action of the chemical constituents of LJF and optimize its pharmaceutical formulations, thereby providing more effective and safer therapeutic options for UC management.

## 8 Mechanistic Insights Into the Anti‐CRC Effects of Bioactive Constituents From LJF

**FIGURE 3:**
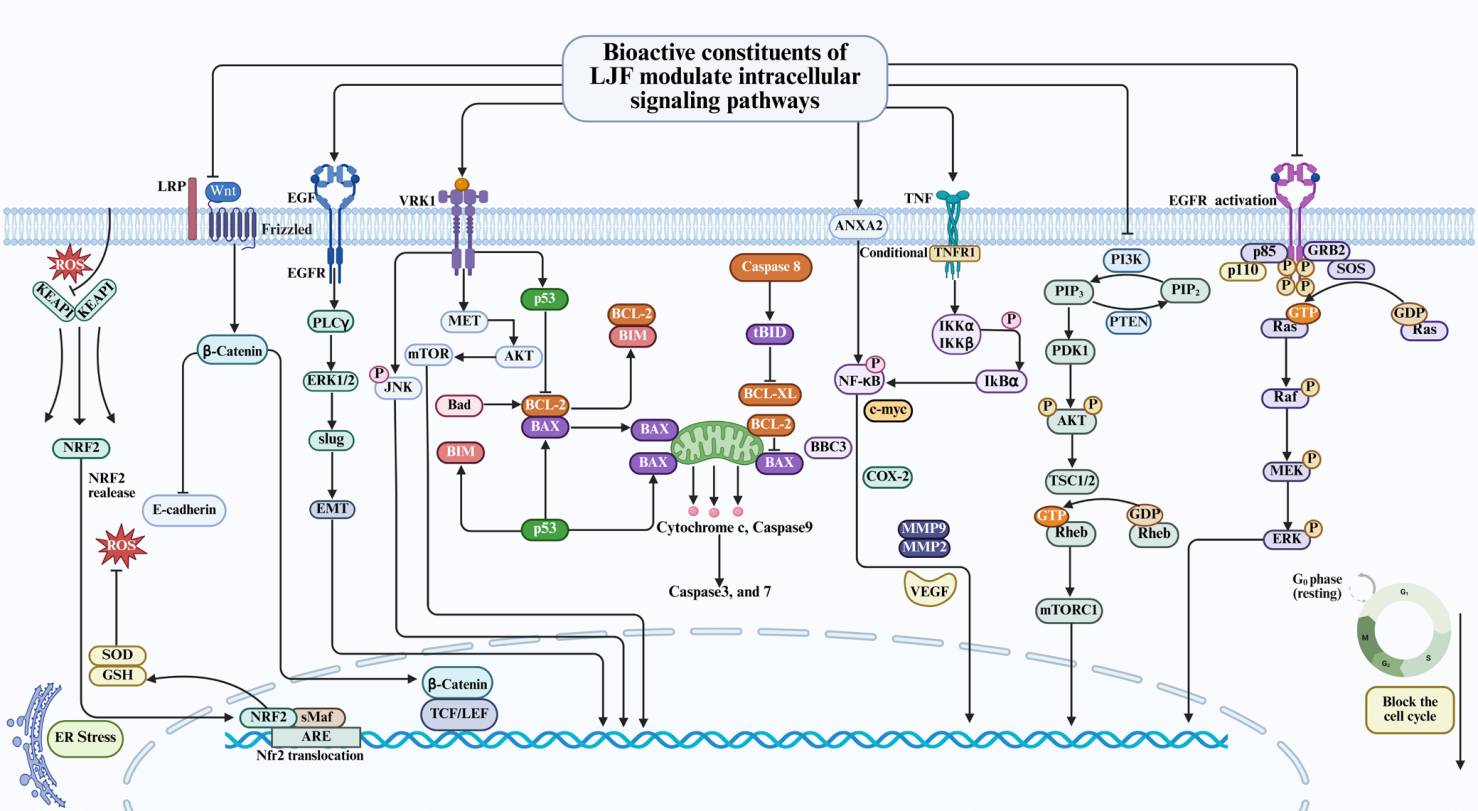
Molecular mechanisms underlying the anti‐CRC effects of bioactive constituents from LJF. These compounds regulate key signaling pathways, including PI3K/Akt, MAPK, NF‐κB, and JAK/STAT, thereby inhibiting cell proliferation, inducing apoptosis and cell‐cycle arrest, and suppressing tumor invasion and metastasis. AKT, protein kinase B; ANXA2, annexin A2; ARE, antioxidant response element; Bad, BCL‐2‐associated agonist of cell death; BAX, BCL‐2‐associated X protein; BBC3/PUMA, BCL‐2‐binding component 3; BCL‐2, B‐cell lymphoma 2; BCL‐XL, B‐cell lymphoma‐extra large; BIM, BCL‐2‐interacting mediator of cell death; Caspase‐3, cysteine‐aspartate protease‐3; Caspase‐7, cysteine‐aspartate protease‐7; Caspase‐8, cysteine‐aspartate protease‐8; Caspase‐9, cysteine‐aspartate protease‐9; c‐Myc, cellular myelocytomatosis oncogene; COX‐2, cyclooxygenase‐2; E‐cadherin, epithelial cadherin; EGFR, epidermal growth factor receptor; EMT, epithelial–mesenchymal transition; ER Stress, endoplasmic reticulum stress; ERK1/2, extracellular signal‐regulated kinase 1/2; GDP, guanosine diphosphate; GRB2, growth factor receptor‐bound protein 2; GTP, Guanosine triphosphate; IκBα, Inhibitor of NF‐κB alpha; KEAP1, Kelch‐like ECH‐associated protein 1; LEF, lymphoid enhancer‐binding factor; LJF, Lonicera japonica Flos; LRP, low‐density lipoprotein receptor‐related protein; MEK, mitogen‐activated protein kinase kinase; MET, mesenchymal‐epithelial transition factor; MMP2, matrix metalloproteinase‐2; MMP9, matrix metalloproteinase‐9; mTOR, mechanistic target of rapamycin; mTORC1, mechanistic target of rapamycin complex 1; NRF2, nuclear factor erythroid 2‐related factor 2; p110, phosphoinositide 3‐kinase catalytic subunit p110; p53, tumor protein p53; p85, phosphoinositide 3‐kinase regulatory subunit p85; PDK1, 3‐phosphoinositide‐dependent protein kinase‐1; PI3K, phosphoinositide 3‐kinase; PIP_2_, phosphatidylinositol 4,5‐bisphosphate; PIP_3_, phosphatidylinositol 3,4,5‐trisphosphate; PLCγ, phospholipase C gamma; PTEN, phosphatase and tensin homolog; Raf, rapidly accelerated fibrosarcoma kinase; Ras, rat sarcoma protein; Rheb, ras homolog enriched in brain; sMaf, small Maf protein; SOS, son of sevenless homolog; tBID, truncated BID; TCF, T‐cell factor; TNF, tumor necrosis factor; TNFR1, tumor necrosis factor receptor 1; TSC1/2, tuberous sclerosis complex 1/2; VEGF, vascular endothelial growth factor; VRK1, vaccinia‐related kinase 1; Wnt, wingless/integrated; β‐Catenin, beta‐catenin.

In recent years, with the steadily increasing incidence and mortality of CRC, identifying potent yet low‐toxicity anti‐CRC agents from natural products has become a major research focus. LJF, a widely used heat‐clearing and detoxifying traditional Chinese medicine, contains a chemically diverse spectrum of constituents, including flavonoids, organic acids, iridoids, and volatile oils. Recent pharmacological studies have indicated that both LJF extracts and various isolated compounds exhibit notable anti‐CRC activity both in vitro and in vivo. For example, Lut inhibits CRC cell proliferation and promotes apoptosis by reducing the expression of cell cycle‐related proteins such as Cyclin D1 and CDK4, activating Caspase‐3/Caspase‐9, and modulating the Bax/Bcl‐2 ratio. Moreover, Lut downregulates MMP‐2 and MMP‐9 expression, thereby impeding cell invasion and metastasis while regulating molecules associated with epithelial‐mesenchymal transition (EMT) (Liu, Guo, et al. 2025). The anti‐CRC mechanisms of LJF encompass multiple layers and signaling pathways, which can be summarized as follows: regulation of the cell cycle to exert antiproliferative effects; induction of tumor cell apoptosis, either directly or synergistically via death receptor pathways or endoplasmic reticulum (ER) stress pathways; and inhibition of tumor cell invasion and metastasis. Specifically, LJF components downregulate the expression and activity of matrix metalloproteinases (MMPs) such as MMP‐2 and MMP‐9, thereby reducing extracellular matrix degradation. Simultaneously, MMPs upregulate adhesion molecules such as E‐cadherin, suppress EMT, and ultimately block CRC cell invasion and distant metastasis. Moreover, modulation of the tumor microenvironment is another potential mechanism underlying the anti‐CRC effects of UC. Bioactive constituents of LJF may regulate inflammatory responses and oxidative stress, thereby improving the tumor microenvironment and suppressing CRC progression (Figure 3).

### 8.1 Inhibition of CRC Cell Proliferation and Induction of Apoptosis

Apoptosis, a tightly regulated form of programmed cell death, plays a pivotal role in maintaining tissue homeostasis. Morphologically, it is characterized by cell shrinkage, chromatin condensation, nuclear fragmentation, and the formation of apoptotic bodies. Under physiological conditions, apoptosis eliminates damaged or aberrantly proliferating cells, thereby preventing tumorigenesis. During cancer progression, however, tumor cells often acquire resistance to apoptosis through the upregulation of antiapoptotic proteins such as Bcl‐2 and Bcl‐xL; the downregulation of proapoptotic factors such as Bax and Bak; or the dysregulation of key signaling pathways including phosphoinositide 3‐kinase/protein kinase B (PI3K/AKT), MAPK, and p53 pathways. Inducing apoptosis remains a central strategy in anticancer therapy, effectively suppressing tumor growth through activation of either the mitochondrial (intrinsic) pathway or the death receptor‐mediated (extrinsic) pathway.

CGA may exert anti‐CRC effects by modulating the cell cycle and mitochondrial apoptotic pathways. In CRC cells, CGA downregulates Bcl‐2 and suppresses NF‐κB signaling while upregulating p21, p53, and caspase‐3/9 and increasing intracellular ROS levels, thereby suppressing cell proliferation and effectively inducing apoptosis (Ranjbary et al. 2023). Studies have also indicated that Kae can induce apoptosis in HT‐29 cells in a dose‐dependent manner. The underlying mechanisms include promoting chromatin condensation and DNA fragmentation, activating caspase‐9, caspase‐3, caspase‐7, and PARP cleavage, enhancing mitochondrial membrane permeability, and facilitating cytochrome C release. Additionally, kaempferol downregulates the antiapoptotic protein Bcl‐xL, upregulates the proapoptotic proteins Bik and Bad, inhibits Akt signaling, and elevates FASL levels to activate caspase‐8. Kae promotes apoptosis in HT‐29 cells through coordinated regulation of both the mitochondrial and death receptor pathways, acting on multiple fronts (Lee et al. 2014). Research has also demonstrated that Lut not only directly inhibits CRC cell proliferation to slow tumor growth but also facilitates apoptosis to eliminate abnormally proliferating cancer cells (Özerkan 2023). Apigenin exerts antitumor effects by inducing apoptosis through the modulation of key molecules such as caspases, BAX, Bcl‐2, and p53 (Mabrouk Zayed et al. 2022).

### 8.2 Induction of Cell Cycle Arrest

Cell cycle arrest refers to halting cell progression at specific phases of the division cycle, such as G0/G1, S, or G2/M, thereby inhibiting cellular proliferation. Numerous bioactive anticancer compounds can achieve this by modulating cyclins and cyclin‐dependent kinases (Cyclins/CDKs) or their inhibitors, effectively preventing cells from advancing to the subsequent phase and ultimately suppressing tumor growth (Glaviano et al. 2025).

CGA exhibits minimal pro‐apoptotic effects on HT‐29 CRC spheroids and does not significantly influence mitochondrial ROS generation. However, at 2000 μM, CGA induces pronounced cell cycle arrest in the G1 phase, suggesting that its anticancer activity in CRC may be mediated through the modulation of the proliferative cycle rather than through the induction of apoptosis or alterations in ROS levels. This G1 arrest may be associated with DNA damage (Vélez et al. 2023). Apigenin can regulate cell cycle progression by inducing arrest at the G0/G1 or G2/M checkpoints (Pandey et al. 2023). Similarly, Que. inhibits CRC cell proliferation by triggering G2/M phase arrest while simultaneously suppressing cell migration (Li, Li, et al. 2025).

### 8.3 Inhibition of Invasion and Metastasis

A hallmark of cancer progression is the ability of CRC cells to migrate and invade surrounding tissues, with EMT playing a pivotal role in tumor metastasis (Vallino et al. 2023).

Results indicate that kaempferol can suppress CRC cell migration in a dose‐dependent manner. Similarly, in a lung metastasis model, kaempferol inhibited the migratory capacity of CRC cells by downregulating circ_0000345 expression, thereby attenuating the activation of the JMJD2C/β‐catenin signaling pathway (Pu et al. 2024). Que. impedes CRC cell invasion and migration through multiple mechanisms, including suppression of EMT, downregulation of MMPs, mitigation of oxidative stress, and modulation of key signaling pathways such as the PI3K/Akt, Wnt/β‐catenin, and JNK pathways (Hsieh et al. 2026). Lut has been reported to suppress the expression or activity of matrix metalloproteinases (MMP‐2 and MMP‐9), which may be associated with reduced extracellular matrix degradation and decreased invasive capacity of colorectal cancer cells (Liu, Guo, et al. 2025).

### 8.4 Modulation of the Tumor Immune Microenvironment

Alterations in cancer cell energy metabolism, particularly the imbalance between glycolysis and mitochondrial oxidative phosphorylation (OXPHOS), can reshape the tumor microenvironment (TME) and influence antitumor immune responses (Jia et al. 2021). Enhancing OXPHOS activity in tumor cells while suppressing excessive glycolysis can decrease lactate production, thereby mitigating the acidic milieu of the TME. This metabolic adjustment not only promotes infiltration of CD8^+^ and CD4^+^ T cells and their secretion of IFN‐γ within tumors, but also may alleviate the immunosuppressive functions of regulatory T cells and myeloid‐derived suppressor cells, collectively amplifying overall antitumor immunity (Jia et al. 2021). However, the reduction in lactate levels in the TME may also restrict lymphocyte metabolism due to insufficient lactate uptake, thereby limiting their OXPHOS activity. Conversely, decreased glycolysis in tumor cells reduces their glucose consumption, which, via the Crabtree effect, increases glucose availability within the TME. This increased glucose supply supports glycolytic metabolism in CD8^+^ T cells, thereby enhancing their effector functions (de Alteriis et al. 2018). It has been reported that extracts of LJF and its bioactive constituent miRNA2911 can suppress the growth of murine colorectal cancer by downregulating TGF‐β1 expression and enhancing T cell infiltration within the tumor, thereby unveiling a potential immunomodulatory mechanism within the tumor microenvironment (Liu et al. 2021).

### 8.5 Modulation of Gut Microbiota and Suppression of Inflammation‐Related Pathways

CAC is a significant complication arising from chronic intestinal inflammation that is frequently observed in patients with IBD. Polyphenolic compounds, which are widely present in the diet, have been shown to modulate the composition of the gut microbiota, thereby alleviating colitis and reducing the risk of CAC development (Zhao and Jiang 2021). These polyphenols can enrich beneficial bacteria, such as *Lactobacillus* and *Bifidobacterium*, while concurrently suppressing proinflammatory microbial populations. The interplay between polyphenol metabolites and the gut microbiota is considered a key protective mechanism underlying their efficacy against colitis and CAC. Among the numerous metabolites in LJF, caffeic acid phenethyl ester (CAPE) is a phenolic acid derivative with notable anti‐inflammatory properties. CAPE can inhibit NLRP3 inflammasome activation, reduce ROS production, and promote NLRP3 ubiquitination, thereby exerting a protective effect against AOM/DSS‐induced colorectal cancer in mice (Dai et al. 2020). These findings suggest that interventions targeting gut microbiota homeostasis may represent a promising strategy for preventing CAC in IBD patients.

In summary, these studies indicate that diverse constituents and metabolites derived from LJF, including polyphenols, can effectively modulate the gut microbiota and inhibit inflammatory pathways, thereby contributing to the management and potential treatment of CAC. Collectively, these findings underscore the potential of LJF as a natural therapeutic agent for the prevention and treatment of CAC in patients with IBD.

### 8.6 Antioxidant Effects

Accumulating evidence indicates that chronic oxidative stress can activate inflammatory signaling pathways, subsequently inducing the expression of various transcription factors or aberrant gene expression, thereby influencing tumor progression and the survival of cancer cells. The Janus kinase (JAK)/signal transducer and activator of transcription (STAT), Wnt/β‐catenin, and PI3K/AKT signaling pathways are closely implicated in CRC pathogenesis due to the deleterious effects triggered by elevated oxidative stress levels (Basak et al. 2020). What's more, oxidative stress induces lipid peroxidation, with various byproducts such as 4‐hydroxy‐2‐nonenal (4‐HNE) and acrolein being strongly associated with CRC progression (Bardelčíková et al. 2023; Lee et al. 2021). Recent investigations have confirmed that apigenin can effectively neutralize ROS, thereby preserving colonic cell integrity to protect against oxidative damage. The antioxidant activity of apigenin is further potentiated by the upregulation of key defense enzymes, including superoxide dismutase (SOD) and catalase, which collectively contribute to a reduced risk of CRC (Fernando et al. 2024).

## 9 Clinical Applications of *Lonicerae Japonicae Flos*

LJF, a traditional medicinal herb with both medicinal and nutritional value, is characterized by a sweet and cold nature and is attributed to the lung, heart, and stomach meridians. It is traditionally acclaimed for its heat‐clearing and detoxifying properties, dispersing wind–heat, and cooling the blood to resolve abscesses, and has a long‐established, broad and reliable foundation for disease prevention and therapy in the clinic. At present, LJF is formulated into a variety of pharmaceutical preparations in clinical practice, including granules, tablets, capsules, oral solutions, injections, and topical formulations.

Shuanghuanglian preparations, composed primarily of LJF, *Scutellaria baicalensis*, and *Forsythia suspensa* , have been developed into multiple pharmaceutical forms, including injections, oral solutions, and granules, and are widely employed in the clinical management of acute respiratory infections and viral diseases (Su et al. 2020; Zhang et al. 2021). In the treatment of pharyngeal inflammation, LJF lozenges are among the commonly used therapeutic agents. LJF dew, a distillate derived from the flower buds of *Lonicera japonica* , is utilized to effectively eliminate internal toxins and attenuate inflammation induced by bacterial infections. In daily life, particularly during the summer months, LJF dew is frequently consumed as a cooling beverage, providing relief from heat‐related discomfort and thirst.

Furthermore, contemporary Chinese patent medicines such as Qingkailing granules consistently retain LJF as a principal constituent within their formulations. When combined synergistically with multiple heat‐clearing, detoxifying, blood‐cooling, and anti‐inflammatory herbs, LJF enhances the overall anti‐inflammatory, antiviral, and immunomodulatory effects of the preparation. Clinically, such compound formulations are frequently employed in the management of febrile illnesses, infectious diseases, and conditions characterized by pronounced inflammatory responses, as they not only alleviate symptoms but also contribute to a reduction in disease duration and the risk of complications.

Throughout the extensive historical evolution of TCM, the principle of “emperor–minister–assistant–courier” has served as a foundational framework for herb compatibility, embodying profound insights into the interactions and synergistic dynamics of medicinal substances (Wu et al. 2023). This principle not only underpins the scientific rationale of multiherb formulations but also provides critical guidance for their clinical application. Within this schema, the emperor herb targets the principal pathomechanism of the disease, exerting the primary therapeutic effect; the minister herb supports and potentiates the action of the Jun herb; the assistant herb functions to harmonize the formula or mitigate toxicity; and the guide herb directs the properties of the herbs to specific meridians, thereby orchestrating a holistic therapeutic outcome. Owing to its integrated heat‐clearing, detoxifying, antimicrobial, and anti‐inflammatory properties, LJF is frequently designated as a Jun herb and is widely applied in clinical practice. Eunkyosan (EKS), known in China as Yinqiao San, is a classical herbal formula centered on LJF and *Forsythia suspensa* (Kim et al. 2023). Originating from *Wen Bing Tiao Bian* and formulated by Wu Tang, EKS represents the earliest prescription for the treatment of warm diseases. With LJF and *Forsythia suspensa* as the Jun herbs, the formula addresses primarily externally contracted wind–heat and the early stage of warm diseases. Clinically, it is extensively utilized to alleviate fever, sore throat, cough, and other symptoms associated with upper respiratory tract infections, underscoring the pivotal role of LJF in early‐stage disease intervention (Fan et al. 2021).

## 10 Dietary Applications of LJF and the Development of Functional Foods

LJF has long been consumed in China and East Asian countries as a dietary therapeutic ingredient, with widespread incorporation into herbal teas and functional beverages (Yang and Yan 2025). Its traditional forms of use include primarily honeysuckle tea, compound cooling herbal infusions, and plant‐based drinks. Historically regarded for its heat‐clearing and detoxifying properties, such empirical use can, within the framework of modern food science and nutrition, be mechanistically reinterpreted as having anti‐inflammatory, antioxidant, and immunomodulatory bioactivities. This translational perspective substantiates its potential as a functional food ingredient in health promotion and disease prevention (Li, Zhang, Yu, et al. 2024).

In recent years, with the growing adoption of the “medicine–food homology” concept, LJF has progressively evolved from traditional decoction forms to diversified food matrices, including instant solid beverages, herbal compound drinks, and functional tea bags. This transition from conventional dietary therapy to modern food formats has not only enhanced convenience and consumer applicability across different settings but also provided a robust foundation for its functional development. As a key functional ingredient, LJF is enriched in flavonoids, phenolic acids, and iridoids, exhibiting promising potential in the regulation of gut health and in the treatment of inflammation‐associated disorders (Ma et al. 2024).

During formulation design and processing, increasing the stability and bioaccessibility of bioactive constituents represents a critical scientific challenge. Given the thermal, photolabile, and oxidation‐sensitive nature of certain compounds in LJF, advanced food processing technologies such as microencapsulation, nanoemulsions, and liposomal delivery systems can be employed to encapsulate and protect the active compounds. Such approaches may effectively minimize degradation during processing and storage while facilitating targeted release and improved intestinal absorption. In addition, the combined application of LJF extracts with prebiotics or probiotics may exert synergistic effects through modulation of the gut microbiota, thereby amplifying the functional benefits in the regulation of gut health.

Moreover, sensory attributes constitute a pivotal determinant of functional food development. The inherent bitterness and astringency of LJF may limit its broader food applications; thus, flavor masking strategies, coformulation with fruit juices or natural sweeteners, and fermentation‐based processing can be utilized to improve palatability while preserving bioactivity and enhancing consumer acceptability. Moreover, the establishment of standardized extraction protocols, robust quality control systems, and well‐defined dose–response relationships is essential to ensure product consistency and support substantiated health claims.

Overall, the incorporation of LJF into functional food systems represents an important translational pathway bridging traditional dietary practices with modern nutritional science. Future studies should further integrate real‐world dietary contexts with in vivo investigations and population‐based intervention trials to validate its long‐term effects on gut health and inflammation‐related biomarkers, thereby advancing its scientific standardization and practical application in the functional food domain.

## 11 Advantages and Challenges of Associated With the Use of LJF Bioactive Components for the Treatment of UC and CRC

### 11.1 Advantages

The bioactive constituents of LJF that alleviate UC symptoms and exert anti‐CRC effects possess multiple notable advantages. Mechanistically, these compounds exhibit multitarget and multipathway synergistic properties, enabling them to modulate diverse biological processes simultaneously (Gao et al. 2019; Li, Zhao, et al. 2021; Nunes et al. 2017). Unlike conventional single‐target pharmaceuticals, the bioactive constituents of LJF that ameliorate UC symptoms act concurrently through multiple pathways, including anti‐inflammatory, antimicrobial, immunomodulatory, and antioxidant mechanisms (Li, Guo, et al. 2023). By suppressing pro‐inflammatory cytokine release, modulating intestinal barrier function, and counteracting oxidative stress simultaneously, these compounds provide comprehensive intervention against the complex pathological processes of UC, minimizing the risk of imbalances associated with single‐target therapies and markedly enhancing therapeutic efficacy (Yan et al. 2020; Zhang et al. 2017). The therapeutic advantage of the bioactive constituents of LJF against CRC lies in their multitargeted profile, low toxicity, and capacity to concurrently modulate inflammation, oxidative stress, the gut microbiota, and multiple oncogenic signaling pathways, thereby conferring a safe and comprehensive antitumor effect.

A defining attribute of LJF is its capacity for systemic homeostatic regulation. Traditional Chinese medicine emphasizes the intrinsic interconnectedness of bodily systems; although UC primarily affects the colon, its pathophysiology is deeply entwined with the overall functional state of the organism. In accordance with TCM principles, LJF exerts its effects by harmonizing the *Yin‐Yang* balance and modulating the functions of various organ systems. In addition to ameliorating local intestinal inflammation and facilitating mucosal repair, LJF orchestrates systemic immune regulation and optimizes metabolic equilibrium. Such multidimensional modulation fortifies host defense mechanisms, mitigates relapse risk, and fosters comprehensive patient recovery (Hu et al. 2019).

Being derived from natural sources, LJF exhibits a favorable safety profile with minimal adverse effects. In contrast to Western therapeutics like glucocorticoids and immunosuppressants, which are often associated with a spectrum of deleterious effects including opportunistic infections, osteoporosis, and hepatotoxicity or nephrotoxicity, LJF, as a traditional Chinese medicinal agent, exhibits a remarkably favorable safety profile at standardized dosages, is exceptionally well tolerated, and benefits from broad patient acceptability.

### 11.2 Challenges

Despite the numerous advantages of the bioactive constituents of LJF in alleviating UC symptoms, several formidable challenges remain to be addressed. The chemical composition of LJF is highly complex, with multiple active compounds interacting synergistically. While this underpins its multitarget therapeutic potential, it also complicates the precise elucidation of individual mechanisms of action and the identification of core efficacious constituents. Moreover, substantial variability in bioactive ingredient content arises from differences in geographical origin, harvest season, and processing method. Standardizing extraction procedures and enriching key bioactive compounds to ensure that LJF preparations have consistent quality and stability represents a critical bottleneck in its modernization and clinical translation.

From a pharmacological perspective, although current studies have provided a certain understanding of the role of LJF in modulating inflammation, antimicrobial activity, immune regulation, oxidative stress, and the gut microbiota, these mechanistic insights remain largely confined to certain key signaling pathways and cytokine profiles. The deeper interconnections among these mechanisms, as well as their spatiotemporal dynamics, have yet to be fully elucidated.

Furthermore, most studies investigating LJF and its bioactive constituents have relied on in vitro or animal models, in which the concentrations or doses used are often considerably greater than those achievable through normal dietary intake. For instance, 2000 μM CGA induced G1‐phase cell cycle arrest in HT‐29 cells, whereas 150 mg/kg LJP modulated the gut microbiota composition in DSS‐induced colitis mice. Although these findings provide valuable insights into the pharmacological potential of LJF‐derived compounds, their physiological relevance under habitual dietary conditions remains uncertain. Therefore, the bioavailability, pharmacokinetics, and actual levels of LJF constituents during human exposure should be considered when their health benefits are interpreted. Future studies should focus on physiologically relevant doses, pharmacokinetic assessments, and well‐designed human intervention trials to validate the health‐promoting effects of LJF and support its evidence‐based development as a functional food.

Clinically, the therapeutic application of the bioactive constituents of LJF for UC and CRC is hampered by the lack of standardized dosing guidelines. The available formulations are diverse and include decoctions, pills, capsules, and enemas, each exhibiting distinct bioavailability and pharmacodynamic profiles, which often creates uncertainty for both clinicians and patients in selecting the most appropriate form. Dosing regimens are often determined empirically or through rudimentary conversions, lacking robust support from large‐scale, multicenter clinical trials. This deficiency increases the risk of subtherapeutic or excessive dosing, potentially compromising efficacy and safety. Treatment duration is often arbitrarily determined, making it difficult to optimize therapeutic outcomes while minimizing relapse risk. There is an urgent need for high‐quality clinical studies to establish evidence‐based protocols, thereby advancing the standardized and scientific application of LJF in the management of UC and CRC.

## 12 Conclusion and Future Perspectives

This study systematically reviewed the potential roles of the bioactive constituents of LJF in UC and CRC. Traditionally, LJF has been employed for heat‐clearing, detoxification, and the management of gastrointestinal discomfort; within the framework of modern food science and nutrition, these effects can be reinterpreted as multifaceted anti‐inflammatory, antioxidant, and immunomodulatory activities. Currently available evidence indicates that LJF is enriched in flavonoids, phenolic acids, and iridoids, which modulate key inflammatory signaling pathways, including the NF‐κB pathway, and regulate gut microbial homeostasis, thereby contributing to the suppression of intestinal inflammation and the modulation of tumorigenesis. Notably, representative compounds such as Lut have been shown to regulate cytokine expression, attenuate inflammatory responses, and improve the gut microbiota composition, underscoring the potential value of LJF in the regulation of gut health. Owing to its multitarget actions, systemic regulatory capacity, and favorable safety profile, LJF has promising prospects for development as a functional food ingredient and dietary supplement.

Nevertheless, several critical challenges remain, including the chemical complexity of its constituents, incomplete mechanistic elucidation, and insufficient standardization, all of which hinder its industrial translation. To address these limitations, integrated multiomics approaches encompassing transcriptomics, proteomics, and metabolomics are warranted to systematically elucidate synergistic interactions among bioactive compounds and their regulatory networks in inflammation‐ and cancer‐related pathways, thereby informing rational formulation design. In terms of processing, the optimization of extraction and purification techniques, such as supercritical fluid extraction, may enhance the stability and enrichment of key constituents. Furthermore, advanced delivery systems, including nanoparticles and liposomes, could improve bioavailability and intestinal targeting, thereby enhancing functional efficacy in food matrices. Rigorous population‐based intervention studies are also needed to evaluate the long‐term effects of LJF or its extracts on intestinal inflammation and metabolic biomarkers under real dietary conditions, providing evidence‐based support for health claims. In addition, exploring synergistic interactions between LJF and other functional ingredients within modern dietary patterns may facilitate the development of multicomponent functional foods and expand its application in the prevention and adjunctive management of chronic inflammatory diseases and gut‐related cancers.

LJF, as a natural resource with a long history of dietary use and for which scientific evidence is growing, holds substantial promise for regulating gut health and preventing disease. Through strengthened mechanistic studies, optimized processing strategies, and robust clinical and nutritional interventions, the transition from a traditional herbal resource into a standardized functional food ingredient is expected.

## Author Contributions

**Xiaoyu Huang:** conceptualization, investigation, software, writing – original draft, visualization. **Letian Zhang:** investigation, visualization, software. **Zenghui Shi:** visualization. **Ni Zhu:** writing – review and editing, methodology. **Yifei Liu:** writing – review and editing, methodology, supervision. **Yanhong Zhou:** supervision, conceptualization, funding acquisition.

## Funding

This study was primarily supported by grants from the 2024 Xianning Municipal Science and Technology Bureau Talent Project (No. 2024RCFW001) and the Research and Innovation Team Project of Hubei University of Science and Technology (No. 2023T09).

## Consent

The authors have nothing to report.

## Conflicts of Interest

The authors declare no conflicts of interest.

## Data Availability

Data sharing not applicable to this article as no datasets were generated or analyzed during the current study.
